# Assessing the factors associated with nurses’ perceptions of decent work: a multicenter cross-sectional study

**DOI:** 10.3389/fpsyg.2026.1807332

**Published:** 2026-04-29

**Authors:** Luoyan Wang, Bowen Xue, Xiaoshan Yang, Jie Zheng, Hong Luo, Yaping Feng

**Affiliations:** 1 Affiliated Mental Health Center and Hangzhou Seventh People’s Hospital, Zhejiang University School of Medicine, Hangzhou, Zhejiang, China; 2 Affiliated Hospital of Hangzhou Normal University, Hangzhou, Zhejiang, China; 3 Department of Psychology and Behavioral Sciences, Zhejiang University, Hangzhou, China; 4 Research Center for Mental Health and Humanities, Zhejiang University School of Medicine, Hangzhou, Zhejiang, China; 5 Hangzhou Geriatric Hospital, Hangzhou, Zhejiang, China; 6 School of Nursing, Shanxi Medical University, Taiyuan, Shanxi, China

**Keywords:** cross-sectional study, decent work, nurses, professional quality of life, social support

## Abstract

**Aim:**

This study aimed to examine factors associated with nurses’ perceptions of decent work and to provide empirical evidence to inform the optimization of their work environment.

**Design:**

This was a multicenter cross-sectional study.

**Methods:**

This study used a convenience sampling method. Between February and March 2024, we recruited a total of 694 clinical nurses from six tertiary hospitals in two provinces of China. The study used the Professional Quality of Life (ProQoL) Scale, the Decent Work Perceptions Scale (DWPS), and the Social Support Rating Scale (SSRS) to collect data. The data were analyzed using descriptive statistics, independent *t*-tests, analysis of variance (ANOVA), Pearson correlation, and stepwise multiple linear regression.

**Results:**

The results showed that social support (*β* = 0.150, *p* < 0.001) and compassion satisfaction (*β* = 0.373, *p* < 0.001) were significantly associated with nurses’ perceptions of decent work. In addition, income (<5,000 vs. 5,000–10,000; <5,000 vs. >15,000), department (*β* = −0.504, *p* < 0.001), job category (*β* = 0.360, *p* = 0.001), and education level (specialist vs. master’s degree) were significantly associated with differences in nurses’ perceptions of decent work (all *p* < 0.05).

**Conclusion:**

The findings indicate that nurses’ perceptions of decent work are related to multiple individual, occupational, and contextual factors. These results suggest that managerial strategies focusing on professional development opportunities, remuneration structures, departmental conditions and work demands, social support, and compassion satisfaction may be relevant to improving nurses’ experiences of decent work.

## 1 Introduction

Nursing is an essential part of the healthcare framework, where the protection of nurses’ work-related rights is pivotal for sustaining the quality of healthcare services, patient safety, and the stable operation of medical institutions (Luther and Hart, 2014). With rapid economic growth, nurses’ expectations of their jobs have transcended mere survival wages; they now expect a favorable work environment, societal recognition, and a sense of professional achievement. However, current research reveals various problems in nursing practice, including excessive workloads, exposure to occupational hazards, low social status, inadequate income, and a lack of sufficient societal support (Rainbow et al., 2024). These issues reduce nurses’ work engagement and job satisfaction, leading to increased burnout, decreased performance, and increased turnover intentions (Rahman, 2020; Waltz et al., 2020; BowenXue et al., 2024; Xue et al., 2024a,b,c). Additionally, decent work significantly influences nurses’ ethical ideologies, job satisfaction, and quality of professional life (Xue et al., 2024a,b,c; Zheng et al., 2024; Zoromba et al., 2025a,b). Therefore, it is essential to explore the various factors that associated with nurses’ perceptions of decent work.

In 1999, the International Labour Organization (ILO) defined “decent work” as encompassing rights at work, employment, social protection, and social dialogue (Brill, 2021). In work psychology theory, Duffy characterizes decent work as comprising five dimensions of individuals’ work life experiences alongside macro conditions: working conditions that guarantee physical and interpersonal safety, sufficient time for rest, organizational values aligned with family and social values, fair compensation, and access to healthcare (Duffy et al., 2016). This viewpoint further enhances the ILO’s definition of decent work. In the context of nursing practice, previous research has indicated that nurses who have a high perception of decent work are better able to adapt to their work environment, preserve their health, and well-being, demonstrate greater enthusiasm and energy for their work, and experience lower rates of burnout and turnover intention (Sönmez et al., 2023; Xue et al., 2024a,b,c). More recent evidence has further suggested that decent work is positively associated with nurses’ vigor at work and work ability (El-Gazar et al., 2025, 2022, 2024a,b,c). Thus, fostering decent work for nurses holds significant value in nursing practice.

Stamm introduced the concept of professional quality of life (ProQoL), which broadly reflects the perceived quality of life among professionals who provide care and support to individuals in need within their work environments (Stamm, 2010a,b). It primarily consists of two components: compassion satisfaction (CS) and compassion fatigue (CF). Compassion satisfaction refers to the fulfillment derived from contributing to meaningful outcomes, supporting one’s organization, and receiving peer support (Wang et al., 2020). Compassion fatigue includes burnout (BO) and secondary traumatic stress (STS). Burnout represents a psychological reaction to chronic workplace stress (Xue et al., 2023), while secondary traumatic stress refers to the adverse emotional reactions that nurses experience when caring for trauma victims (Missouridou, 2017). Studies have demonstrated that factors such as the work environment, compensation, and work hours play pivotal roles in determining the quality of professional life (Adolfo, 2021). For nurses, these factors constitute everyday working conditions that shape their experiences of providing compassionate care. Higher levels of ProQoL are indicative of more supportive work environments and are associated with greater job satisfaction, lower turnover intention, and more favorable patient-related outcomes. Recent international studies have continued to show that nurses’ compassion satisfaction, burnout, and secondary traumatic stress are closely related to work-related and psychosocial factors, such as job satisfaction, sleep quality, work engagement, and intent to leave (Mansouri et al., 2025; Rayani et al., 2024; Saliya et al., 2024; Wei et al., 2024). Taken together, these findings suggest that ProQoL is not only shaped by nurses’ working conditions, but may also be relevant to how they evaluate the dignity, support, and quality of their work experiences. These patterns are consistent with the core principles of decent work (Cruz et al., 2020; Niu et al., 2022). Accordingly, compassion satisfaction, burnout, and secondary traumatic stress represent key dimensions of ProQoL that are closely associated with nurses’ perceptions of decent work.

Social support refers to the diverse forms of help and resources that individuals gain from their social networks, ranging from tangible assistance offered by family, friends, and colleagues to the intangible sense of care, respect, and belonging fostered within these relationships (Cao et al., 2021; Zhang et al., 2020). Workplace social support has been found to predict positive outcomes and mitigate the impact of adverse events (Harding et al., 2022). According to Duffy, sufficient social support can significantly influence an individual’s perception of decent work (Duffy et al., 2016). Similarly, other studies have revealed a relationship between social support and the way individuals view decent work (Su and Wong, 2023; Wang et al., 2019). One possible explanation is that social support may enhance nurses’ sense of recognition, belonging, and psychological safety, while also increasing their access to interpersonal and organizational resources (Hirvikallio et al., 2024; Zheng et al., 2024). In demanding clinical environments, such support may help buffer the negative effects of work stress and strengthen nurses’ perceptions that their work is respected, protected, and meaningfully supported (Li et al., 2025; Pietrzak et al., 2025). From the perspective of the JD-R model, social support can be understood as a key job resource that may improve how nurses evaluate the quality, fairness, and sustainability of their work (Duffy et al., 2016; Zheng et al., 2024). As a result, social support represents an important contextual factor associated with nurses’ perceptions of decent work.

The Job Demands-Resources (JD-R) model provides a useful theoretical framework for understanding factors associated with nurses’ perceptions of decent work. According to the JD-R model, job demands refer to aspects of work that require sustained physical or psychological effort and are associated with physiological and psychological costs, whereas job resources refer to aspects of work that help employees achieve work goals, reduce the impact of job demands, and support growth and development (Brough et al., 2013). In the context of nursing, night shift frequency and work in demanding clinical departments may reflect elevated job demands, while social support, more stable employment conditions, and more favorable income conditions may reflect access to job resources. Recent studies in nursing have continued to support the relevance of the JD-R model in explaining how work demands and resources are associated with nurses’ burnout, well-being, and work-related outcomes (Li et al., 2025; El-Gazar et al., 2025). Burnout and secondary traumatic stress may be understood as strain-related experiences linked to the health-impairment process, whereas compassion satisfaction may reflect a positive motivational experience supported by meaningful and resourceful work conditions. Importantly, nurses’ perceptions of decent work may be understood as reflecting how they evaluate whether their work is adequately supported, fairly rewarded, developmentally meaningful, and sustainable under existing job demands and resources. From this perspective, the JD-R model provides a conceptual basis for examining how occupational context, social support, and professional quality of life are associated with nurses’ perceptions of decent work.

Previous research has explored the relationships between nurses’ perceptions of decent work and several work-related variables, including organizational support, work–family enrichment, burnout, job satisfaction, and turnover intention (Xu and Zhao, 2024; Xue et al., 2024a,b,c). In addition, studies have suggested that social support is positively associated with more favorable perceptions of decent work (Song and Lee, 2023). Nevertheless, empirical evidence on the factors associated with nurses’ perceptions of decent work remains limited, particularly regarding the combined roles of professional quality of life and social support. Furthermore, most existing studies have focused on a relatively narrow set of predictors, and evidence from multicenter nursing samples is still insufficient. The present study addresses these gaps by examining occupational, contextual, and psychosocial factors associated with nurses’ perceptions of decent work in a multicenter sample of nurses in China. It also contributes to the literature by interpreting these associations through the Job Demands-Resources model and the Psychology of Working Theory.

Based on the JD-R model and previous literature, this study proposed the following hypotheses: H1, social support is positively associated with nurses’ perceptions of decent work; H2, compassion satisfaction is positively associated with nurses’ perceptions of decent work; H3, burnout is negatively associated with nurses’ perceptions of decent work; and H4, secondary traumatic stress is negatively associated with nurses’ perceptions of decent work. In addition, demographic and occupational characteristics were examined as exploratory contextual factors associated with nurses’ perceptions of decent work.

## 2 Methods

### 2.1 Design

This study used a cross-sectional study design. Data collection occurred from February to March 2024. The reporting of the study adheres to the Strengthening Reporting of Observational Studies in Epidemiology (STROBE) guidelines (Cevallos and Egger, 2014).

### 2.2 Participants

This study used a convenience sampling method to recruit participants from six Grade A tertiary hospitals in two provinces of China. The hospitals had an average of 1,500 beds and a bed occupancy rate of 94%. Convenience sampling was adopted because the study was conducted in busy clinical settings across multiple hospitals, where probability-based sampling was not feasible within the available study period and staffing conditions. This approach facilitated the recruitment of a sufficient number of eligible nurses from different departments within a limited timeframe. The participating hospitals were all tertiary hospitals, and the sample was intended to reflect nurses working in these participating hospitals rather than the broader nursing population in China. Therefore, the findings should be interpreted within this sampling scope.

Inclusion criteria were as follows: (1) registered nurses; (2) had more than 1 year of clinical experience; (3) were on duty during the survey; and (4) voluntary participation. Exclusion criteria: were as follows: (1) nurses in management or logistics; (2) had a history of psychiatric illness; and (3) refused to participate.

Among the 842 nurses who completed the survey, 694 valid questionnaires remained after those with anomalous responses, such as identical repetitive items or excessively short response times, were discarded, resulting in an effective response rate of 82.42%.

### 2.3 Sample size

Sample size calculations were performed via G*Power (version 3.1) software, opting for a linear multiple regression approach with a set medium effect size of 0.15, an *α* of 0.05, and a power (1−*β* error probability) of 0.95. The investigation included 22 variables, including 11 sociodemographic characteristics and 11 dimensions related to scales. The calculated minimum sample size required was 226 nurses. To account for a projected 10% rate of invalid questionnaires, a minimum of 249 nurses were deemed necessary for participation in the study.

### 2.4 Instruments

#### 2.4.1 Demographic characteristics

The demographic characteristics of the nurses included gender, age, educational background, years of working, professional title, department, marital status, with or without children, monthly income, type of employment, and frequency of night shifts per month.

#### 2.4.2 Decent work

In this study, the Decent Work Perceptions Scale was used to assess nurses’ perceptions of decent work. The scale was developed by Chinese scholars and has been applied in Chinese occupational and nursing research, demonstrating acceptable psychometric properties in these contexts (Mao et al., 2014; BowenXue et al., 2024). The scale comprises five dimensions: work rewards, work position, work atmosphere, work development, and work recognition, with a total of 16 items. The responses to these items are scored via a 5-point Likert scale, where 1 means “strongly disagree” and 5 means “strongly agree.” The aggregate score ranges from 16 to 80, with higher scores indicating a stronger perception of decent work. The DWPS demonstrated high internal consistency in this study, with a Cronbach’s *α* coefficient of 0.946.

#### 2.4.3 Professional quality of life

This study used the professional quality of life (ProQoL) scale to measure nurses’ levels of compassion satisfaction, burnout, and secondary traumatic stress. The ProQoL Scale was originally developed by Stamm and has been widely used in healthcare research. In the present study, we used the Chinese version that has been applied in Chinese nursing populations and has demonstrated acceptable reliability and validity (Stamm, 2010a,b; Niu et al., 2022). The scale consists of three dimensions: compassion satisfaction, burnout, and secondary traumatic stress. It includes 30 items rated on a 5-point Likert scale ranging from 1 (“never”) to 5 (“always”), with items 1, 4, 15, 17, and 29 reverse-scored. Each dimension is scored independently. In this study, the Cronbach’s *α* coefficients for compassion satisfaction, burnout, and secondary traumatic stress were 0.935, 0.815, and 0.856, respectively.

#### 2.4.4 Social support

This study used the Social Support Rating Scale to measure the level of social support among nurses. The scale was developed by Chinese scholars and has been widely used in Chinese populations, including nursing samples, with acceptable reliability and validity reported in previous studies (Xiao et al., 1991; Shen et al., 2022). The scale consists of 10 items divided into three subscales: objective support (3 items), subjective support (4 items), and support utilization (3 items). Items 1–4 and 8–10 are scored using a single-selection format, with options 1, 2, 3, and 4 corresponding to scores of 1, 2, 3, and 4 points, respectively. Item 5 is divided into five parts (A, B, C, D, E), with scores ranging from 1 to 4 points for each part, ranging from “none” (1 point) to “full support” (4 points). Items 6 and 7 are scored as 0 if the response is “no sources” and are assigned points corresponding to the number of sources indicated. A higher aggregate score indicates greater perceived social support. The Cronbach’s α coefficient for the scale was 0.736 in this study.

### 2.5 Data collection

This study used an online method for data collection. Initially, a dedicated research team was established to convert all questionnaires into online links, ensuring their functionality. The questionnaire consisted of three parts: the first part included informed consent, directing nurses who chose to decline participation to the end; the second part introduced the purpose, significance, potential risks, and contact information of the study; and the third part comprised the questionnaire content. Members responsible for data collection, who were from the hospitals’ nursing management departments, received uniform training on survey administration. They then distributed the questionnaire links to nurses, explained the study purpose and completion instructions, and provided clarification when needed. To reduce potential common method bias, the survey was administered anonymously with standardized instructions, and duplicate submissions were restricted by limiting each account, device, and IP address to a single response.

### 2.6 Data analysis

This study used IBM SPSS Statistics version 26.0 for statistical analysis. Descriptive statistics were used to summarize the demographic characteristics of the participants, with categorical variables presented as percentages and normally distributed continuous variables reported as means ± standard deviations. *Q*–*Q* plots and Levene’s test were applied to assess the normality and homogeneity of variance of the variables. For variables that met assumptions of normality and homogeneity, independent-sample *t*-tests or one-way analysis of variance (ANOVA) were conducted to compare differences in decent work perception scores across groups. Pearson correlation analysis was performed to examine the associations between study variables and decent work perceptions. A stepwise multiple linear regression analysis was then conducted with the total decent work perception score as the dependent variable. This approach was used as an exploratory method to identify variables that remained statistically associated with decent work perceptions after accounting for other measured factors. Variables that were statistically significant in univariate analyses (*p* < 0.05) and showed associations with decent work perceptions were entered as independent variables. Because several predictors, particularly psychosocial variables such as social support, compassion satisfaction, burnout, and secondary traumatic stress, may be conceptually and statistically interrelated, multicollinearity was also assessed in the regression analysis. Variance inflation factor (VIF) values were calculated to evaluate multicollinearity among the predictors. Cronbach’s *α* coefficient was used to assess the internal consistency of the scales. A two-sided *p* value of less than 0.05 was considered statistically significant.

### 2.7 Ethical consideration

This study was approved by the Ethics Committee of the Second Hospital of Shanxi Medical University (Approval No. 2024-038). Nurses participating in the study were fully informed about the principle of voluntary participation and were assured that they could withdraw at any time without any consequences. In accordance with research ethics involving human participants, all data in this study were stored in secure electronic folders managed by authorized research personnel, with access restricted to the researchers involved in this study. Informed consent was obtained electronically from all participants before questionnaire completion.

## 3 Results

### 3.1 Demographic characteristics

Table 1 shows that the average age of the nurses who participated in the study was 33.34 ± 6.74 years. The vast majority of nurses are female, accounting for 96.8% of the sample. In terms of education, 87.9% of the nurses held a bachelor’s degree. The most common range of work experience is 5 to 15 years, encompassing 54% of the sample. Most nurses were married (77.4%) and without children (57.1%). Over half of the nurses had a monthly income between 5,000 and 10,000 CNY, which constituted 53.2% of the participants. The majority of nurses are temporarily employed, accounting for 70.3% of all nurses. Regarding departments, a significant proportion of nurses work in medicine or surgery departments (69.9%). Finally, 43.8% of the nurses reported having 5–7 night shifts per month.

**Table 1:** Demographic characteristics of nurses (*N* = 694).

Characteristics	Category	Frequency	Percentage	Decent work		
				M ± SD	*t*/*F*	*p*
Age				33.34 ± 6.74		
Gender^a^	Male	22	3.2%	51.32 ± 11.78	−0.351	0.726
	Female	672	96.8%	52.65 ± 12.36		
Educational background^b^	Associate degree or lower	55	7.9%	48.60 ± 10.90	9.928	<0.001^c^
	Bachelor’s degree	610	87.9%	52.14 ± 12.05		
	Master’s degree or higher	29	4.2%	60.97 ± 16.38		
Years of working^b^	<5 years	196	28.2%	51.10 ± 13.56	1.159	0.325
	5–15 years	375	54%	52.87 ± 11.56		
	16–25 years	75	10.8%	52.84 ± 12.02		
	>25 years	48	6.9%	50.81 ± 13.32		
Professional title^b^	Nurse	82	11.8%	50.57 ± 12.06	1.284	0.279
	Senior nurse	240	34.6%	51.79 ± 13.30		
	Supervisor nurses	323	46.5%	52.62 ± 11.54		
	Cochief nurses or above	49	7.1%	54.55 ± 12.73		
Marital status^b^	Married	537	77.4%	51.70 ± 12.38	2.681	0.069
	Single	150	21.6%	54.23 ± 12.01		
	Other	7	1.0%	49.29 ± 12.54		
With or without children^a^	Without	396	57.1%	52.94 ± 12.50	1.759	0.079
	With	298	42.9%	51.28 ± 12.06		
Monthly income (CNY)^b^	<5,000	243	35.0%	49.35 ± 12.01	19.438	<0.001^d^
	5,000–10,000	369	53.2%	52.55 ± 11.39		
	10,001–15,000	43	6.2%	54.51 ± 10.84		
	>15,000	39	5.6%	64.56 ± 15.81		
Job category^a^	Permanently employed	206	29.7%	56.53 ± 13.55	5.703	<0.001^**^
	Temporarily employed	488	70.3%	50.41 ± 11.31		
Department^a^	Medicine or surgery	485	69.9%	49.91 ± 11.10	7.315	<0.001^**^
	Specialty Units	209	30.1%	57.60 ± 13.34		
Night shifts per month^b^	≤4	246	35.4%	54.22 ± 11.86	9.357	<0.001 ^e^
	5–7	304	43.8%	52.29 ± 11.82		
	≥8	144	20.7%	48.69 ± 13.43		

^**^p < 0.001; ^a^Independent sample *t*-test; ^b^One-way ANOVA test; ^c^Post-hoc test: Associate degree or lower vs. Bachelor (*p* = 0.039), Associate degree or lower vs. Master or higher (*p* < 0.001), Bachelor vs. Master or higher (*p* < 0.001); ^d^Post-hoc test:<5,000 vs. 5,000–10,000 (*p* = 0.001), <5,000 vs. 10,001–15,000 (*p* = 0.009), <5,000 vs. >15,000 (*p* < 0.001), 5,000–10,000 vs. >15,000 (*p* < 0.001), 10,001–15,000 vs. >15,000 (*p* < 0.001); ^e^ Post-hoc test:≤4 vs. ≥8 (*p* < 0.001), 5–7 vs. ≥8 (*p* = 0.004).

### 3.2 Descriptive analyses

The results revealed that nurses had an average decent work score of 52.23 ± 12.33. The average compassion satisfaction score was 25.82 ± 6.74, whereas the average burnout score was 29.62 ± 7.06. The average secondary trauma score was 27.62 ± 7.69. The average social support score was 40.76 ± 7.27. See Table 2.

**Table 2:** Correlation of social support, compassion satisfaction, burnout, STS, and decent work.

Variables	Mean	SD	Social support	Compassion satisfaction	Burnout	STS	Decent work
Social support	40.76	7.27	1				
Compassion satisfaction	25.82	6.74	0.344^**^	1			
Burnout	29.62	7.06	−0.120^**^	−0.126^**^	1		
STS	27.62	7.69	−0.205^**^	−0.515^**^	0.346^**^	1	
Decent work	52.23	12.33	0.324^**^	0.479^**^	−0.148^**^	−0.272^**^	1

^**^p < 0.01; SD, Standard Deviation; STS, Secondary Traumatic Stress.

### 3.3 Univariable analysis

In addition, as shown in Table 1, nurses’ perceptions of decent work differed significantly according to educational background (*t* = 9.928, *p* < 0.001), monthly income (*F* = 19.438, *p* < 0.001), job category (*t* = 5.703, *p* < 0.001), department (*t* = 7.315, *p* < 0.001), and frequency of night shifts (*F* = 9.357, *p* < 0.001).

### 3.4 Correlations among variables

The results of the correlation analysis indicate that social support is positively correlated with compassion satisfaction (*r* = 0.344) and decent work (*r* = 0.324), whereas it is negatively correlated with burnout (*r* = −0.120) and secondary traumatic stress (*r* = −0.205). Compassion satisfaction was negatively correlated with burnout (*r* = −0.126) and secondary traumatic stress (*r* = −0.515). Additionally, burnout and secondary traumatic stress were positively correlated (*r* = 0.346). Decent work was negatively correlated with both burnout (*r* = −0.148) and secondary traumatic stress (*r* = −0.272), whereas it was positively correlated with Compassion satisfaction (*r* = 0.479).

### 3.5 Multiple linear regression analysis

Decent work perception scores were treated as the dependent variable in the multiple linear regression analysis. Education level, monthly income, job category, department, monthly night shift frequency, social support, burnout, secondary traumatic stress, and compassion satisfaction were entered as independent variables.

In Step 1, monthly income (<5,000 CNY vs. >15,000 CNY, *β* = −0.200, *p* < 0.001), job category (*β* = 0.475, *p* < 0.001), department (*β* = −0.655, *p* < 0.001), educational background (associate degree or lower vs. master’s degree or higher, *β* = −0.095, *p* = 0.031), and night shift frequency (≤4 vs. 5–7, *β* = 0.093, *p* = 0.028) showed statistically significant associations with nurses’ decent work perception scores. This model accounted for 14.9% of the variance.

In Step 2, the inclusion of social support increased the explained variance to 22.2%. After accounting for the demographic variables, social support was significantly associated with decent work perception scores (*β* = 0.275, *p* < 0.001). In this step, monthly income (<5,000 vs. >15,000, *β* = −0.171, *p* < 0.001), job category (*β* = 0.462, *p* < 0.001), educational background (associate degree or lower vs. master’s degree or higher, *β* = −0.091, *p* = 0.033), and department (*β* = −0.622, *p* < 0.001) remained statistically significant, whereas night shift frequency (≤4 vs. 5–7, *β* = 0.078, *p* = 0.052) was no longer significant.

In Step 3, adding burnout increased the explained variance to 22.9%. Burnout showed a statistically significant negative association with decent work perception scores (*β* = −0.090, *p* = 0.009).

In Step 4, the inclusion of secondary traumatic stress further increased the explained variance to 25.3%. Secondary traumatic stress was negatively associated with decent work perception scores (*β* = −0.171, *p* < 0.001). In this model, social support (*β* = 0.240, *p* < 0.001), monthly income (<5,000 vs. >15,000, *β* = −0.168, *p* < 0.001), job category (*β* = 0.426, *p* < 0.001), and department (*β* = −0.583, *p* < 0.001) remained statistically significant. Educational background (associate degree or lower vs. master’s degree or higher, *β* = −0.079, *p* = 0.056) and burnout (*β* = −0.035, *p* = 0.320) were no longer significant.

In Step 5, compassion satisfaction was added to the model, increasing the explained variance to 34.4%. Compassion satisfaction showed a statistically significant positive association with decent work perception scores (*β* = 0.373, *p* < 0.001). In addition, social support (*β* = 0.150, *p* < 0.001), monthly income (<5,000 vs. >15,000, *β* = −0.157, *p* < 0.001), job category (*β* = 0.360, *p* = 0.001), and department (*β* = −0.504, *p* < 0.001) remained statistically significant. Education level (specialist vs. master’s degree, *β* = −0.084, *p* = 0.031) and monthly income (<5,000 vs. 5,000–10,000, *β* = −0.082, *p* = 0.023) were also significantly associated with decent work perception scores.

Overall, the adjusted *R*^2^ increased from 14.9% in Step 1 to 34.4% in Step 5, indicating that the variables included in the model accounted for a substantial proportion of the variance in nurses’ perceptions of decent work. The increase in explained variance across the models suggests that psychosocial and experiential factors added important explanatory value beyond demographic and occupational characteristics. The multivariable findings provided support for H1 and H2, while H3 and H4 were supported in the bivariate analyses but not in the final regression model. Multicollinearity diagnostics indicated that the VIF values for the predictors ranged from 1.17 to 1.68, suggesting that multicollinearity was not a serious concern in the regression analysis. The detailed results are presented in Table 3.

**Table 3:** Multiple linear regression for variables.

Variables	Step 1				Step 2				Step 3				Step 4				Step 5			
	*β*	SE	*t*	*p*	*β*	SE	*t*	*p*	*β*	SE	*t*	*p*	*β*	SE	*t*	*p*	*β*	SE	*t*	*p*
Job category	0.475	3.235	3.961	<0.001	0.462	3.094	4.026	<0.001	0.458	3.081	4.01	<0.001	0.426	3.038	3.783	<0.001	0.360	2.853	3.402	0.001
Department	−0.655	3.249	−5.415	<0.001	−0.622	3.109	−5.376	<0.001	−0.612	3.097	−5.31	<0.001	−0.583	3.053	−5.129	<0.001	−0.504	2.869	−4.719	<0.001
Associate degree or lower vs. bachelor	−0.032	1.713	−0.709	0.479	−0.029	1.638	−0.68	0.497	−0.027	1.631	−0.632	0.528	−0.028	1.606	−0.655	0.513	−0.045	1.506	−1.125	0.261
Associate degree or lower vs. master or higher	−0.095	2.726	−2.157	0.031	−0.091	2.607	−2.142	0.033	−0.083	2.601	−1.977	0.048	−0.079	2.561	−1.911	0.056	−0.084	2.4	−2.168	0.031
<5,000 vs. 5,000–10,000	−0.082	1.004	−2.015	0.044	−0.073	0.961	−1.878	0.061	−0.071	0.957	−1.837	0.067	−0.058	0.944	−1.516	0.13	−0.082	0.887	−2.287	0.023
<5,000 vs. 10,001–15,000	−0.050	1.955	−1.317	0.188	−0.056	1.869	−1.519	0.129	−0.061	1.864	−1.667	0.096	−0.053	1.837	−1.461	0.144	−0.056	1.721	−1.651	0.099
<5,000 vs. >15,000	−0.200	2.05	−5.208	<0.001	−0.171	1.97	−4.634	<0.001	−0.175	1.964	−4.775	<0.001	−0.168	1.935	−4.655	<0.001	−0.157	1.814	−4.619	<0.001
≤4 vs. 5–7	0.093	1.275	2.207	0.028	0.078	1.221	1.946	0.052	0.073	1.217	1.811	0.071	0.063	1.2	1.598	0.111	0.027	1.13	0.734	0.463
≤4 vs. ≥8	0.032	1.023	0.774	0.439	−0.002	0.984	−0.049	0.961	−0.008	0.981	−0.214	0.831	−0.023	0.969	−0.585	0.559	−0.031	0.908	−0.856	0.392
Social support					0.275	0.058	8.063	<0.001	0.266	0.058	7.779	<0.001	0.240	0.058	7.042	<0.001	0.150	0.056	4.518	<0.001
Burnout									−0.090	0.059	−2.637	0.009	−0.035	0.062	−0.996	0.320	−0.064	0.058	−1.914	0.056
STS													−0.171	0.058	−4.774	<0.001	0.009	0.061	0.234	0.815
Compassion satisfaction																	0.373	0.07	9.778	<0.001
*R*^2^	0.160				0.233				0.241				0.265				0.356			
Adjusted *R*^2^	0.149				0.222				0.229				0.253				0.344			

STS, Secondary Traumatic Stress; SE, Standard error.

## 4 Discussion

This study identified several factors associated with nurses’ perceptions of decent work, including education level, monthly income, job category, department, social support, and compassion satisfaction.

The mean decent work score among nurses was 52.23 ± 12.33, indicating a moderate level of perceived decent work. This finding is broadly consistent with previous studies reporting moderate levels among nurses, including studies with scores of 52.94 ± 6.46 and 52.46 ± 14.05 (Yu et al., 2024; Zheng et al., 2024), while remaining slightly lower than that reported among psychiatric nurses (55.69 ± 10.42) (Xue et al., 2024b). One possible explanation is that nurses in general clinical settings often face substantial emotional labor, burnout, and work-related stress, which may contribute to less favorable evaluations of work conditions (Gulsen and Ozmen, 2020; Xue et al., 2024a,b,c). In contrast, psychiatric nurses may receive more specialized training in emotional regulation, crisis intervention, and violence prevention, which may partly account for their relatively higher perceptions of decent work. These findings suggest that work context and professional preparation may be important in understanding differences in decent work perceptions across hospital settings.

Education level was significantly associated with nurses’ perceptions of decent work, with nurses who had higher educational attainment reporting more favorable perceptions. This pattern differs from some previous nursing research (Cao et al., 2025), but may reflect the stronger professional identity, broader knowledge, and greater confidence often associated with higher education (Neishabouri et al., 2017). This interpretation should be made cautiously because the number of nurses with master’s degrees or higher in the present sample was relatively small.

Monthly income was also significantly associated with decent work perceptions. Previous research on living wages suggests that income is relevant not only to basic subsistence but also to fairness, quality of life, and the broader meaning of work (Carr et al., 2016; Searle and McWha-Hermann, 2021; Yao et al., 2017). From the perspective of the JD-R model, income may therefore be understood as an important material and organizational resource associated with nurses’ perceptions of decent work. Job category was also significantly associated with decent work, with temporarily employed nurses reporting lower levels of decent work than permanently employed nurses. This pattern is consistent with the Psychology of Working Theory, which highlights workers’ needs for security and stability (Duffy et al., 2016). Temporary nurses may face greater uncertainty, fewer benefits, and more limited opportunities for professional development, all of which may weaken their sense of security and organizational recognition (Peel and Inkson, 2004; Connell and Burgess, 2006; Varasteh et al., 2022). In JD-R terms, such conditions may reflect insufficient access to key job resources.

Department was also significantly associated with nurses’ perceptions of decent work. Nurses in specialty units, such as emergency departments and intensive care units, reported higher decent work scores, possibly because these settings offer stronger professional autonomy, clearer role meaning, and more opportunities for advanced skill use and teamwork (Stark et al., 2023; Adriaenssens et al., 2011; Galletta et al., 2016). By contrast, nurses with more frequent night shifts reported lower levels of decent work in univariable analyses. Night shift work has been associated with sleep disruption, poorer recovery, and lower job satisfaction (Okechukwu et al., 2022; Knap et al., 2022), suggesting that demanding schedules may negatively shape work evaluations. Although night shift frequency was not retained in the final regression model, this pattern still indicates that demanding work arrangements may be relevant to nurses’ perceptions of decent work.

Social support was significantly associated with nurses’ perceptions of decent work. This is consistent with previous research highlighting the relevance of social support to decent work perceptions (Song and Lee, 2023). Support from organizations, colleagues, family members, and the wider social environment may enhance nurses’ recognition, psychological safety, and sense of professional value (Zhan et al., 2020; El-Gazar and Zoromba, 2024). Social support has also been linked to lower work-related strain and higher job satisfaction (Wu et al., 2021; Orgambídez et al., 2022). Within the JD-R framework, social support can be understood as a key job resource that helps nurses manage demanding work conditions and maintain more positive work experiences, which may explain why it remained significant in the final model. This may also be broadly consistent with evidence from other service-sector settings suggesting that organizational support can help sustain positive work-related outcomes under changing workplace conditions (Edeh et al., 2025a,b).

Compassion satisfaction was also significantly associated with decent work perceptions and showed the strongest association in the final model. In nursing practice, work often involves sustained emotional engagement with vulnerable patients, and these experiences may either promote fulfillment or contribute to strain. Higher compassion satisfaction may reflect nurses’ sense of accomplishment, professional value, and enjoyment derived from helping others effectively (Stamm et al., 2010; Sacco et al., 2015). Within the JD-R framework, compassion satisfaction may reflect the motivational pathway through which meaningful work experiences and adequate resources contribute to more favorable evaluations of work. This may help explain why compassion satisfaction was such a strong correlate of decent work in the present study (Zheng et al., 2024).

Although burnout and secondary traumatic stress were negatively correlated with decent work in the bivariate analyses, they were not retained in the final regression model. Secondary traumatic stress may reflect the emotional strain associated with repeated exposure to patients’ suffering and trauma, which may in turn be linked to less favorable evaluations of the work environment and lower perceptions of decent work. The fact that burnout and secondary traumatic stress were not retained in the final model may indicate that their explanatory role overlaps with other psychosocial factors, particularly those related to support and positive professional experiences (Ayed et al., 2024; Shaqiqi and Abou El-Soud, 2024). Nevertheless, the negative bivariate findings suggest that these strain-related experiences remain relevant to understanding nurses’ work experiences and warrant further study.

From a theoretical perspective, the present findings extend the application of both the Psychology of Working Theory and the Job Demands-Resources framework to nurses’ perceptions of decent work. The results suggest that decent work perceptions are shaped not only by structural and employment-related conditions, but also by psychosocial resources and positive professional experiences. In this sense, the findings highlight the importance of support, security, and meaningful work in understanding how nurses evaluate the quality and dignity of their work (Duffy et al., 2016; El-Gazar et al., 2025).

### 4.1 Implications

The findings of this study have several practical implications for nursing management and workforce policy. First, because social support was positively associated with nurses’ perceptions of decent work, hospital administrators may consider strengthening peer support, supervisory support, and team-based communication mechanisms. Beyond interpersonal support, broader organizational conditions may also matter. Attention to organizational culture may be important, as a supportive organizational culture may help individuals adapt more positively to demanding and changing work conditions (Edeh et al., 2025a,b). Likewise, fostering organizational learning may also be valuable, as learning-oriented environments may support resilience and adaptation under changing work conditions (Edeh et al., 2024). In practice, these goals may be advanced through structured mentoring systems, regular team debriefings in high-stress units, and supportive leadership practices, which may help nurses feel more respected, protected, and valued in their work environments and may also contribute to more psychologically safe team climates (Jangland et al., 2021; El-Gazar et al., 2024a,b,c).

Second, compassion satisfaction was also positively associated with decent work perceptions, suggesting that managerial strategies should not focus solely on reducing burnout, but also on enhancing positive professional experiences. Recognition of nurses’ contributions, opportunities for reflective practice or case-sharing, and feedback on patient improvement and care outcomes may help strengthen compassion satisfaction and reinforce the meaning and value of patient care (Sacco et al., 2015).

Third, the findings related to income, job category, and department suggest that organizational and structural conditions remain highly relevant to nurses’ perceptions of decent work. Hospitals may therefore consider reviewing compensation fairness, reducing inequities between temporary and permanent nurses in access to benefits, training, and promotion opportunities, and adopting department-specific staffing and scheduling strategies for units with heavier workload intensity or more frequent night shifts (Hidayah and Ananda, 2021; Li et al., 2024). In addition, differentiated professional development pathways and continuing education opportunities may be particularly important for nurses at different educational levels and career stages (Jangland et al., 2021).

At the policy level, healthcare institutions and nursing managers may also consider strengthening employment protection, promoting more equitable access to organizational resources, and developing workforce policies that better address workload intensity, shift-related demands, and access to professional development (Li et al., 2024). Overall, the findings suggest that improving nurses’ perceptions of decent work requires not only individual-level support, but also organizational and policy-level efforts to create fairer, more supportive, and more sustainable working conditions.

### 4.2 Limitations

This study has several limitations that should be acknowledged. First, its cross-sectional design precludes causal inference regarding the relationships among social support, professional quality of life, occupational factors, and decent work perceptions. Future longitudinal, prospective, intervention-based, or quasi-experimental studies are needed to clarify temporal ordering and directionality and to better examine whether changes in work-related support and resources are associated with subsequent changes in nurses’ perceptions of decent work. Second, the use of convenience sampling and the inclusion of nurses from tertiary hospitals in only two provinces of China may limit the generalizability of the findings to other regions, hospital levels, and healthcare systems. Third, because all variables were measured through an online self-report questionnaire at a single time point, common method bias and social desirability bias cannot be fully excluded. In addition, the findings were based on self-reported perceptions rather than objective organizational indicators or external assessments. Future research would benefit from combining self-report data with supervisor ratings, administrative records, or qualitative interviews. Finally, some potentially important organizational factors, such as staffing adequacy, leadership style, workload intensity, and institutional support policies, were not measured. Although the JD-R model provided a useful interpretive framework, the full set of JD-R pathways was not tested. Future studies could address these issues using broader organizational measures and theory-driven analytic approaches, such as longitudinal modeling or structural equation modeling.

## 5 Conclusion

In summary, this study examined factors associated with nurses’ perceptions of decent work and found that education level, monthly income, job category, department, social support, and compassion satisfaction were significantly associated with decent work perceptions. These findings contribute empirical evidence to the understanding of how individual, occupational, and contextual characteristics relate to nurses’ experiences of decent work. The results also suggest that employment arrangements, compensation structures, departmental conditions, social support, and compassion satisfaction may be particularly relevant when considering nurses’ perceptions of decent work. Such considerations may help inform future discussions on nursing management and policy development, particularly in relation to workplace dignity and supportive work environments.

## Data Availability

The raw data supporting the conclusions of this article will be made available by the authors, without undue reservation.
